# Activation of the EGFR/ERK pathway in high-grade mucoepidermoid carcinomas of the salivary glands

**DOI:** 10.1038/sj.bjc.6605788

**Published:** 2010-07-27

**Authors:** B Lujan, S Hakim, S Moyano, A Nadal, M Caballero, A Diaz, A Valera, M Carrera, A Cardesa, L Alos

**Affiliations:** 1 Department of Pathology, Hospital Clínic, IDIBAPS, University of Barcelona, Villarroel, 170, Barcelona 08036, Spain; 2 Department of Otolaryngology, Hospital Clínic, University of Barcelona, Villarroel, 170, Barcelona 08036, Spain; 3 Department of Pathology, Hospital Universitari de Bellvitge, Feixa Llarga, Hospitalet de Llobregat, Barcelona 08907, Spain

**Keywords:** mucoepidermoid carcinoma, salivary gland carcinoma, epidermal growth factor, EGFR, extracellular signal-regulated kinase, ERK1/2

## Abstract

**Background:**

Mucoepidermoid carcinoma (MEC) shows differences in biological behaviour depending mainly on its histological grade. High-grade tumours usually have an aggressive biological course and they require additional oncological treatment after surgery.

**Methods:**

In a series of 43 MECs of the salivary glands, we studied the epidermal growth factor receptor (EGFR) gene by using dual-colour chromogenic *in situ* hybridisation (CISH). Moreover, we assessed the protein expressions of the EGFR and the activated extracellular signal-regulated kinases (pERK1/2) by using immunohistochemistry. These results were correlated with the histological grade of the tumours and the outcome of the patients.

**Results:**

The CISH study demonstrated a high-EGFR gene copy number, with balanced chromosome 7 polysomy, in 8 out of 11 high-grade MECs (72.7%), whereas 27 low-grade and 15 intermediate-grade tumours had a normal EGFR gene copy number (*P*<0.001). The EGFR gene gains correlated with disease-free interval (*P*=0.003) and overall survival of the patients (*P*=0.019). The EGFR protein expression had a significant correlation with the histological grade of the tumours but not with the outcome of the patients. The pERK1/2 expression correlated with histological grade of tumours (*P*<0.001), disease-free interval (*P*=0.004) and overall survival (*P*=0.001).

**Conclusions:**

The EGFR/ERK pathway is activated in high-grade MECs with aggressive behaviour. Patients with these tumours who require oncological treatment in addition to surgery could benefit from EGFR and mitogen-activated protein kinase pathway inhibitors.

Mucoepidermoid carcinoma (MEC) is the most frequent malignant tumour that originates in the major and minor salivary glands, and represents about one third of all malignant salivary gland tumours (Spiro, 1986; Goode and El-Naggar, 2005; Ellis and Auclair, 2008). It is a heterogeneous neoplasm that may present different biological behaviour, depending mainly on the histological grade of the tumour (Goode *et al*, 1998; Goode and El-Naggar, 2005; Ellis and Auclair, 2008; Nance *et al*, 2008). Surgical resection is the standard treatment for all grades of MEC. Radiotherapy after wide surgical excision of the tumour is recommended for high-grade MECs. Lymphadenectomy and adjunvant external beam radiotherapy are indicated when cervical metastases are present. Chemotherapy is indicated in the treatment of metastatic disease and in the palliation of locoregional disease not amenable to either salvage surgery or radiation therapy (Agulnik and Siu, 2004; Nance *et al*, 2008). Low-grade MECs usually do not recur; most patients are cured after surgery and the 5-year survival rate is 76–95%. Conversely, high-grade MECs are aggressive neoplasms that frequently have an infiltrative pattern of growth, recur and even metastasize, and their 5-year survival rate is 30–50% (Goode *et al*, 1998; Nance *et al*, 2008).

To date, clinical trials using targeted therapies on salivary gland tumours are scarce, probably because of the low number of these cases in each institution. Only one phase II study of Herceptin (Trastuzumab) with disappointing results in patients with advanced salivary gland tumours overexpressing HER2/neu has been published (Haddad *et al*, 2003). Incomplete clinical trials using epidermal growth factor receptor (EGFR) antagonists have been performed on patients with salivary gland cancer. Moreover, studies on the oncogenetic pathways in salivary gland MECs predictive of response to targeted therapies are scarce and incomplete. In recent years, strategies against the EGFR family and the mitogen-activated protein kinase (MAPK) signalling pathway have received special attention in the treatment of cancer. The EGFR family, including the four distinct receptors EGFR/ErbB1, HER2/cerbB2, HER3/CerbB3 and HER4/ErbB4, has been identified as a potential therapeutic target in solid tumours. The EGFR/ErbB1 is a gene located on chromosome 7p12 and has emerged as a significant factor in the development and growth of many types of cancer, playing an important role in cancer-cell proliferation, angiogenesis and metastasis. This gene encodes a 170-kDa membrane glycoprotein that can be activated by phosphorylation and induce a downstream signalling transduction cascade. A major signalling route of the EGFR is the Ras-Raf-MAPK pathway (Klapper *et al*, 2000). Activation of Ras initiates a multistep phosphorylation cascade that leads to the activation of MAPKs. The MAPK extracellular signal-regulated kinases ERK1/2 are the best characterised and are most strongly associated with human cancer. The ERK1/2 are activated by dual phosphorylation on a tyrosine and a threonine residue by dual-specificity kinases, and subsequently regulate cell transcription and have been linked to proliferation, survival and transformation (Lewis *et al*, 1998).

The EGFR antagonists are included in treatment protocols of advanced stages of non-small cell carcinoma of the lung, colorectal cancer and head and neck squamous cell carcinoma (Ciardello and Tortora, 2008). In head and neck tumours, EGFR can be abnormally activated and protein overexpression by the neoplastic cells is frequently detected by immunohistochemistry (Nicholson *et al*, 2001; Grandis and Sok, 2004). However, EGFR amplifications are not frequent and EGFR activating mutations are very rare (Kalyankrishna and Grandis, 2006). The EGFR overexpression has been correlated with poor prognosis in head and neck cancer (Kalyankrishna and Grandis, 2006). To date, several clinical trials have been carried out to identify the molecular characteristics of the tumours predictive of response to EGFR antagonists. The EGFR activating mutations and increased EGFR gene copy number identify the most sensitive population in these tumours (Cappuzzo *et al*, 2005; Hirsch *et al*, 2005; Tsao *et al*, 2005; Sartore-Bianchi *et al*, 2007). In most MECs of the salivary gland, the EGFR protein is overexpressed (Gibbons *et al*, 2001; Shang *et al*, 2007), but EGFR activating mutations are extremely rare (Han *et al*, 2008; Dahse and Kosmehl, 2008; Dahse *et al*, 2009). However, studies on the EGFR gene copy number have not been performed before in a series of salivary gland MECs.

In our previous studies, we saw that high-grade MECs with aggressive course differ molecularly from low-grade tumours. These high-grade tumours overexpress the oncogenic glycoprotein MUC1 (Alos *et al*, 2005; Handra-Luca *et al*, 2005). MUC1 acts as a proto-oncogene that interacts with EGFR and correlates with MAPK activation in mouse models (Schroeder *et al*, 2001) and inhibits the ligand-mediated ubiquitinisation and degradation of EGFR *in vitro* (Pochampalli *et al*, 2007). Moreover, the expression of ERK1/2 MAPKs has been related to aggressive tumour behaviour in MECs of the salivary glands (Handra-Luca *et al*, 2003). We therefore hypothesised that histological high-grade MECs, which have a clinically aggressive course, may harbour EGFR protein overexpression and high-EGFR gene copies linked to aggressive tumour biology. To investigate this, we studied the EGFR gene by using chromogenic *in situ* hybridisation (CISH) with a dual-colour probe, in a series of 43 MECs. This new technique obtains the same results as fluorescence *in situ* hybridisation (FISH) and offers potential advantages over FISH to detect gene copy number, including the ability to distinguish between areas of tumour and normal tissue.

In addition to genetic analysis, the immunohistochemical study of the EGFR protein was performed and activated ERK1/2 were assessed by using an antibody specific for the dually phosphorylated and activated ERK1 and ERK2 (MAPK phospho-p44/42). These molecular studies have been correlated with the histological characteristics of the tumours and the follow-up of the patients.

## Materials and methods

### Selection of cases

Forty-three MECs diagnosed at the Department of Pathology of the Hospital Clinic, and Hospital Princeps d’Espanya, Bellvitge, University of Barcelona, from 1996 until 2005, were reviewed. The medical records were obtained from patients’ files in the Departments of Otorhinolaryngology and Maxillofacial Surgery. The study was approved by the Local Ethical Committee and patients gave their informed consent. At diagnosis, the tumours were staged according to the American Joint Committee on Cancer (Sobin and Wittekind, 2002). All patients underwent primary surgery as standard treatment. Lymph node dissection was performed only in cases with lymph node metastases. Full-dose radiotherapy was applied after tumour excision with positive margins, when lymph node metastases were assessed, and in locoregional recurrences. Chemotherapy with cisplatin was added for palliative purposes, in patients with lymph node metastases (N2 or N3) and in cases with tumoural persistence after surgery and resistance to radiotherapy.

### Histological grading of MECs

Haematoxylin-eosin and alcian blue-stained slides and paraffin wax-embedded material were available for all cases. The MECs were graded following the 2005 World Health Organization Classification of Tumours (Goode and El-Naggar, 2005).

### CISH and immunohistochemistry

Representative paraffin wax blocks were selected from each of the 43 cases for CISH and immunohistochemistry.

The CISH was performed on a 4-*μ*m section of each tumour that was deparaffined in two changes of xylene and three washes of degraded ethanol for 3 min each. The slides were pretreated with CISH pretreatment buffer (Dako, Carpinteria, CA, USA) and heated to 92°C, and then rinsed with distilled water. The tissues were digested for 10 min with pepsin digestion solution (Dako) at room temperature, washed twice in distilled water for 5 min each, dehydrated in 70, 85 and 96% alcohol for 2 min each and dried. A measure of 10 *μ*l of dual-colour EGFR Spectrum-red/CEP7 Spectrum-blue probe (Dako) were applied to each slide. Sections were covered with coverslips and denatured on a hot plate at 82°C for 5 min. Hybridisation was done overnight at 37°C. Then the slides were washed in 2 × SSC at 73°C for 2 min and three times in distilled water. Then the sections were blocked with H_2_O_2_ in absolute methanol and incubated with a blocking reagent for 10 min at room temperature. The hybridisation signals were detected after sequential incubations with anti-mouse anti-DIG (60 min at room temperature), polymerised horseradish peroxidase anti-mouse antibody (60 min) and 3,3-diaminobenzedine (DAB). The sections were counterstained with haematoxylin.

Immunohistochemical studies were carried out using the automated immunohistochemical system TechMate 500 (Dako), and the EnVision system (Dako). Briefly, 4 *μ*m sections were deparaffinised and hydrated using graded alcohols and water. For antigen retrieval, an autoclave pretreatment at 120°C for 5 min was performed. Peroxidase was blocked for 7.5 min in ChemMate peroxidase-blocking solution (Dako). The slides were incubated with the primary antibodies for 30 min and washed in ChemMate buffer solution (Dako). The peroxidase labelled polymer was then applied for 30 min. After being washed in ChemMate buffer solution, the slides were incubated with DAB substrate chromogen solution, washed in water, counterstained with haematoxylin, washed, dehydrated and mounted. The primary antibodies used in the study were: EGFR (Dako; dilution 1 : 100) and pERK1/2 (Phospho-p44/42; Thr202/Tyr204) (Cell Signaling Technology, Beverly, MA, USA; dilution 1 : 50). Appropriate positive and negative controls were used.

The CISH and immunohistochemical results were evaluated by two independent observers (BL and LA). For CISH evaluation, a light microscope under a × 40 objective was used. A total number of 100 tumoural cells were evaluated. The centromeric blue signal and the EGFR red signal in each cell were counted and the proportion centromeric/EGFR signal number was calculated. The cases were considered normal if two blue and two red signals were visualised in each nuclear cell. Polysomy was considered when ⩾3 blue and red signals (in equal number) were seen in each nucleus. The EGFR amplification was defined as red signals >1.5 blue signals.

The immunostain for EGFR was evaluated: 0, no positive cells; 1+, low discontinuous membrane staining; 2+, unequivocal membrane staining with moderate intensity and 3+, strong and complete membrane staining. Only cases with 2+ and 3+ staining patterns were considered positive. The pERK1/2 showed nuclear positivity. For analytical purposes, positivity for EGFR and pERK1/2 was considered when ⩾10% of tumour cells were positive. High-pERK1/2 expression was considered when ⩾30% of positive cells were detected.

### Statistical analysis

The continuous clinical variables considered were follow-up and age (median and range were calculated). Overall survival was calculated from diagnosis to the death of the patient or loss of follow-up. Disease-free interval was the time from surgical excision of the tumour to the first recurrence or metastasis. Both overall survival and disease-free interval were analysed by the Kaplan–Meier method. The categorical clinical variables were gender (female/male), location of tumours (parotid/submaxillary/minor salivary gland) and stage (I/II/III/IV). The categorical histological variables considered were histological grade (1/2/3) and molecular results: EGFR protein expression (positive/negative), EGFR gene copy (normal/polysomy) and ERK1/2 expression (>30% of positive cells/<30% of positive cells). Fisher's exact test was used for comparison between qualitative variables and Student's *t*-test and ANOVA were applied for quantitative variables according to the application conditions. All tests were two sided. Differences were analysed by the log-rank method. Differences were considered to be statistically significant with an *α* risk of 0.05.

## Results

### Clinicopathological characteristics of the patients

The clinicopathological characteristics of the patients at diagnosis, the treatment details and outcome are summarised in Table 1.

After a median follow-up of 62 months, 33 (76.8%), 6 (13.9%) and 4 (9.3%) patients were alive and disease free, alive with disease and died of disease, respectively. The median disease-free interval was 96 months (range 0–159 months). Relapses occurred in 19 (44.1%) patients: in 14 (32.6%) patients, a local tumoural recurrence took place, and in 5 (11.6%) patients, there was lymph node metastasis.

The statistical associations of the disease-free interval and overall survival with histological grade of tumours and molecular results are expressed in Table 2. Patients with high-grade tumours had shorter disease-free interval (*P*=0.001) and overall survival (*P*=0.001) than those with low- and intermediate-grade tumours.

### EGFR gene analysis

**Figure 1:**
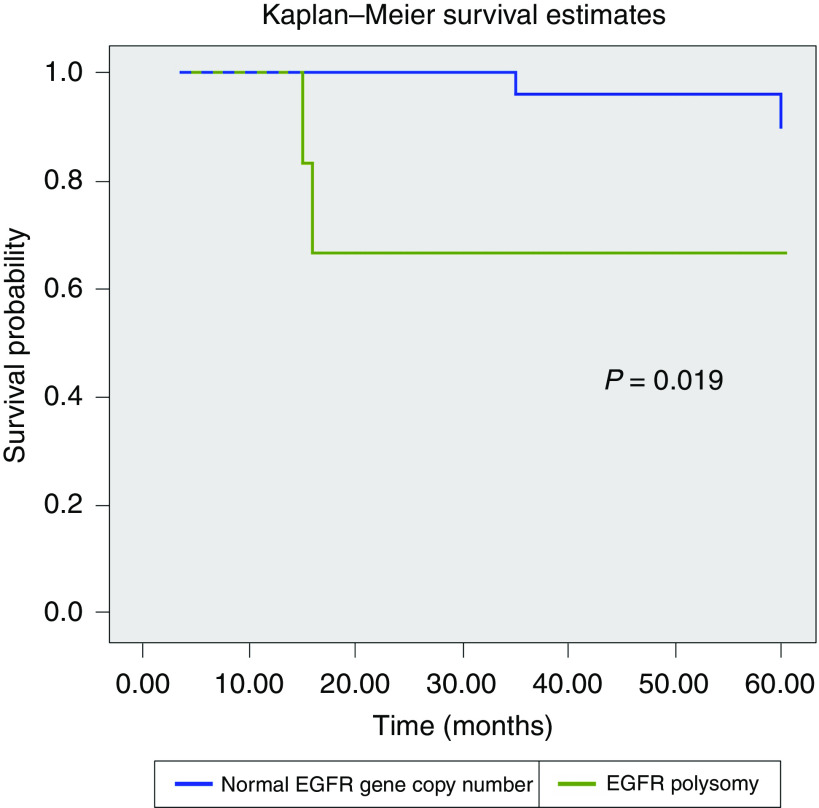
Kaplan–Meier curve for overall survival stratified by CISH results (normal EGFR gene copy number *vs* increased EGFR gene copy number).

Eight cases (18.6%) had chromosome 7 polysomy. In these cases, there were >2 signals of both centromere and EGFR signals in over 70% of cells, but the relationship between both signals was 1 : 1. In two cases there were 3 signals (low polysomy) and in six cases there were >3 signals (high polysomy). No cases with EGFR amplification were detected. All of the eight cases with chromosome 7 polysomy were high-grade MECs, whereas the rest of the tumours (27 low grade, 5 intermediate grade and 3 high grade) showed a normal pattern of expression (*P*<0.001). Chromosome 7 polysomy was associated with shorter disease-free interval (*P*=0.003) and overall survival (*P*=0.019) (Figure 1).

### EGFR and pERK1/2 protein expression

The EGFR protein expression was positive in 34 tumours (79%). All cases with chromosome 7 polysomy showed expression of the EGFR protein (*P*<0.001). These cases showed positivity in >60% of tumoural cells. High-EGFR protein expression was associated with high-histological grade of tumour (*P*<0.001, ANOVA), but it was associated with neither disease-free interval (*P*=0.286) nor overall survival (*P*=0.307).

**Figure 2:**
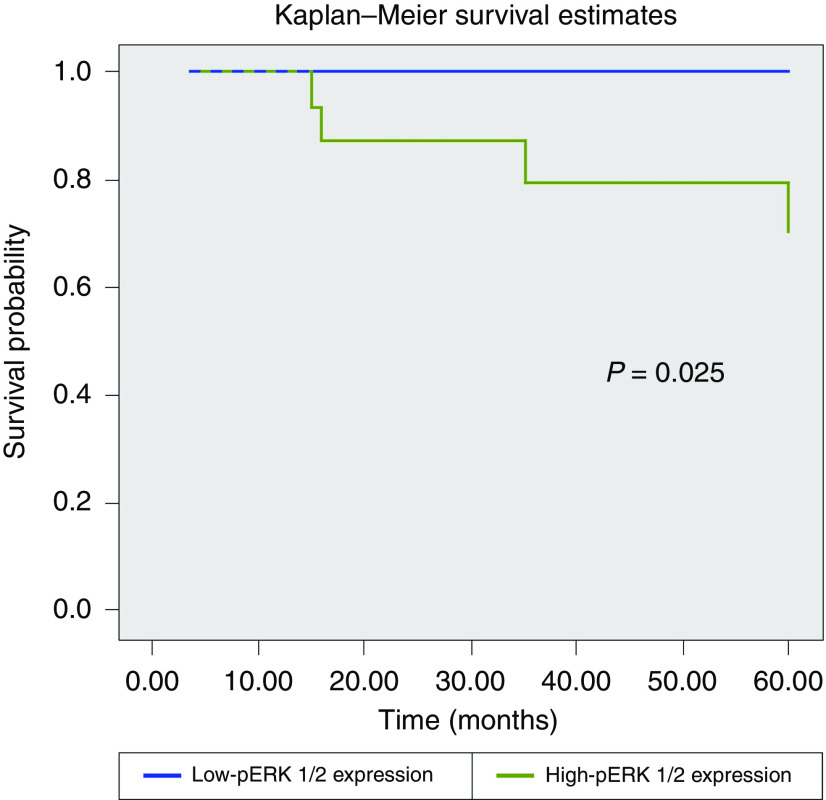
Kaplan–Meier curve for overall survival stratified by activated ERK1/2 expression (low-pERK1/2 expression *vs* high-pERK1/2 expression).

**Figure 3:**
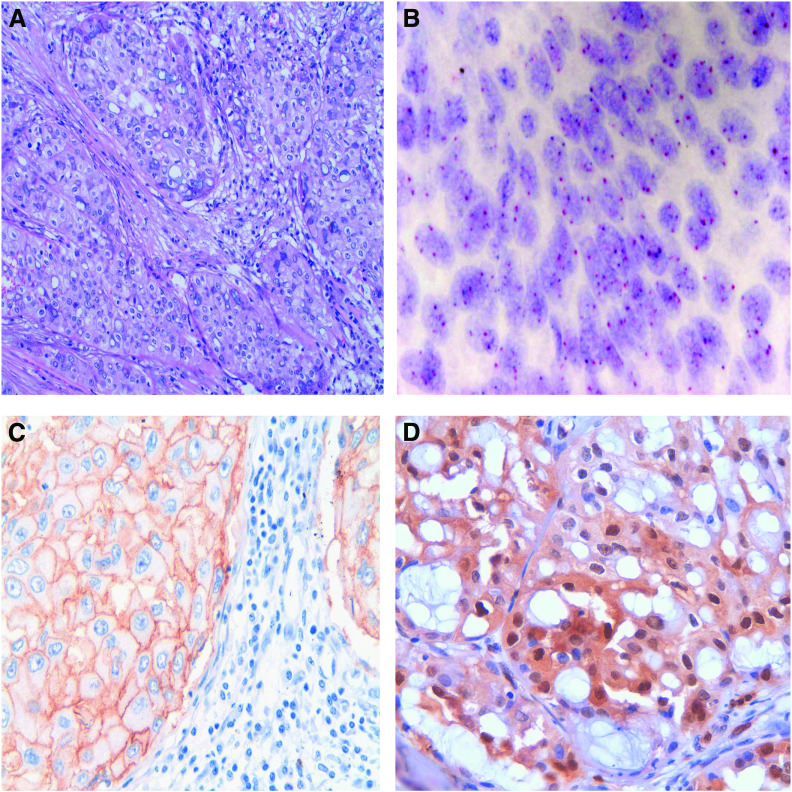
An example of high-grade mucoepidermoid carcinoma. (**A**) Histological characteristics of the neoplasm (HE × 200). (**B**) The CISH analysis shows high polysomy. Four or five signals (both red EGFR and blue centromere) are seen in each nucleus in most of the neoplastic cells (EGFR CISH × 630). (**C**) Expression of EGFR protein with strong and diffuse membrane positivity (EGFR × 400). (**D**) High expression of activated ERK1/2 with nuclear positivity in most of the neoplastic cells (pERK1/2 × 400).

The pERK1/2 protein was expressed in 34 tumours (79%). There was a statistical correlation between pERK1/2 positivity and histological grade of tumour (*P*<0.001, ANOVA), shorter disease-free interval (*P*=0.004) and overall survival (*P*=0.001). High-pERK1/2 expression (positivity in ⩾30% of neoplastic cells) was observed in 21 tumours (49%). High-pERK1/2 expression was associated with shorter overall survival (*P*=0.025) (Figure 2), but not with disease-free interval (*P*=0.108). All cases with EGFR polysomy had high expression of pERK1/2 (*P*=0.002) and there was a marginally significant correlation between high expression of pERK1/2 and EGFR immunohistochemical expression (*P*=0.047) (Figure 3).

## Discussion

This study shows that high-grade MECs with aggressive behaviour harbour an increased EGFR gene copy number and high expression of pERK1/2 MAPKs. In spite of the fact that EGFR amplification was not seen in any of the 43 cases of this series, in six of them there was high polysomy with ⩾4 EGFR gene copies. The EGFR gene is rarely amplified in human cancers, but the increased EGFR gene copy number with balanced chromosome 7 polysomy in cancer cells is relatively frequent, in ∼24–40% of patients with non-small cell lung cancer, squamous-cell carcinoma of the head and neck or colorectal cancer. Chromosome 7 polysomy has been linked to tumour aggressiveness and poor clinical outcome (Hirsch *et al*, 2003; Ciardello and Tortora, 2008). In this study, all cases with EGFR gene gains had a significant shorter disease-free interval and overall survival. The EGFR product is a membrane glycoprotein composed of an extracellular ligand-binding domain, a transmembrane lipophilic component and an intracellular protein kinase domain. The ligand binding induces EGFR dimerisation, activation of the intrinsic tyrosine kinase protein and tyrosine phosphorylation with the activation of a cascade of biochemical and physiological responses (Lewis *et al*, 1998). This downstream signalling transduction activates MAPKs through phosphorylation by MAPK kinases, and the activation of this pathway is associated with cell proliferation and oncogenic transformation (Grandis and Sok, 2004). In this series, there was a significant correlation between increased EGFR copy number and high expression of pERK1/2 (*P*=0.002). High expression of activated ERK1/2 has been related to tumour progression in several neoplasms (Albanell *et al*, 2001; Adeyinka *et al*, 2002) and in salivary gland MECs (Handra-Luca *et al*, 2003). In this series, the pERK1/2 expression was significantly correlated with shorter disease-free interval and overall survival. Furthermore, MAPKs can also be activated through the upstream activation of HER2/neu or RAS. About one third of salivary gland MECs have HER2/neu gene amplification (Press *et al*, 1994) and about one fifth of MECs harbour H-RAS mutations (Yoo and Robinson, 2000a), but K-RAS mutations are extremely rare (Yoo and Robinson, 2000b). However, to define the molecular mechanisms underlying the biological behaviour in high-grade MECs, *in vitro* experiments with cell lines should be carried out.

The immunohistochemical expression of EGFR in the majority of MECs that we have observed is concordant with other studies (Gibbons *et al*, 2001; Shang *et al*, 2007). All cases with chromosome 7 polysomy had an expression of EGFR protein of over 60% of cells. Nevertheless, most immunohistochemical positive cases failed to show an increased EGFR gene copy number. This discrepancy between the EGFR gene copy number and the immunohistochemical detection of the protein has been reported before in several cancers, and has been attributed to a post-transcriptional phenomenon mediated at the mRNA level (Grandis and Tweardy, 1993). There was a significant correlation between the EGFR protein expression and the histological grade of the tumours, but not with the clinical outcome of the patients. The current grading system classification is three-tiered, and tumours are classified into low-, intermediate- and high-grade MECs, depending on the architecture and cellular characteristics of the neoplasms. Low-grade tumours are usually well-defined tumours, often cystic with a predominance of mucous cells, whereas high-grade MECs usually have an infiltrative pattern of growth, are solid and mainly composed of intermediate type and epidermoid cells. High mitotic index, cellular anaplasia, necrosis and perineural invasion are characteristics of high-grade tumours (Spiro, 1986; Ellis and Auclair, 2008). Significant differences in the outcome of the patients related to histological grade have been repeatedly confirmed in series of MECs of the salivary glands (Goode *et al*, 1998; Alos *et al*, 2005; Nance *et al*, 2008). In this study, a statistical correlation between the histological grade and disease-free interval and overall survival of the patients was found. The prognostic value of the EGFR polysomy, and the EGFR and the pERK1/2 protein expressions were related to the histological grade.

Strategies against EGFR include monoclonal antibodies able to bind to the extracellular domain of the receptor such as cetuximab, or small molecule ATP-competitive tyrosine kinase inhibitors (TKIs), such as gefitinib and erlotinib. Some clinical, histopathological and molecular characteristics have been proposed for identifying the population sensitive to EGFR-TKI treatment in non-small carcinoma of the lung (Sone *et al*, 2007). Activating mutation in exons 18, 19 and 21 of the EGFR gene has proved to be a significant factor in predicting response to EGFR-TKIs in non-small cell carcinoma of the lung. However, these mutations are less common in the United States and European population than in the Asian population (Sone *et al*, 2007), and data from large randomised studies indicate that increased EGFR gene copy number is probably the best factor in predicting response and evaluate overall survival of the patients (Hirsch *et al*, 2005; Tsao *et al*, 2005; Cappuzzo *et al*, 2005). Interestingly, a good response to EGFR antagonists in head and neck and lung carcinomas with expression of MAPKs has been observed (Albanell *et al*, 2001; Gandara *et al*, 2004). Moreover, immunohistochemical positivity for activated ERK1/2 has been correlated with a good response to MAPKs inhibitors in clinical trials on cutaneous melanomas (Jilaveanu *et al*, 2009). Therefore, the high-grade MECs in this series, with increased EGFR gene copy number and pERK1/2 high expression could be sensitive to EGFR or MAPKs antagonists.

The MECs of the lung share histological and molecular characteristics with salivary gland MECs. Some series on lung MECs have shown lack of EGFR mutations in these tumours and a percentage of chromosome 7 polysomy of 17%, similar to the results in our series (Macarenco *et al*, 2008). However, some lung MECs have been described as having activating EGFR mutations in the Asian population (Han *et al*, 2008). The MECs and adenosquamous carcinomas share histological characteristics and differential diagnosis between both tumour types may be challenging in the head and neck region and lung (Alos *et al*, 2004; Rossi *et al*, 2009). Adenosquamous carcinomas are aggressive tumours arising from upper or lower airways, whereas MECs have a salivary or bronchial gland origin, whose prognosis depends on the histological grade. Adenosquamous carcinomas usually harbour EGFR activating mutations, whereas MECs do not (Kang *et al*, 2007; Han *et al*, 2008; Macarenco *et al*, 2008; Rossi *et al*, 2009). Previous studies on salivary gland MECs have found that EGFR mutations are extremely rare (Han *et al*, 2008; Dahse and Kosmehl, 2008; Dahse *et al*, 2009).

To date, few cases on metastatic salivary gland MECs with EGFR gene gains with chromsome 7 polysomy and good response to EGFR monoclonal antibody cetuximab have been published (Grisanti *et al*, 2008). However, clinical trials that include a large series of salivary gland MECs are difficult to carry out because of the low number of these cases in each institution.

In conclusion, we have identified that high-grade salivary gland MECs usually have an increased EGFR gene copy number and highly express pERK1/2. The activation of the EGFR/ERK pathway in these tumours is associated with aggressive behaviour and could represent potential indicators of response to EGFR antagonists or MAPK pathway inhibitors.

## Figures and Tables

**Table 1:** Clinicopathological characteristics of the patients at diagnosis, treatment details and outcome

**Characteristic**	**No. of cases (%)**
Patient number	43
	
*Age (years)*	
Median (range)	53 (4–82)
	
*Gender*	
Female	23 (53.5%)
Male	20 (46.5%)
	
*Tumour location*	
Parotid gland	22 (51%)
Minor salivary gland	15 (35%)
Submaxillary gland	6 (14%)
	
*Histological grade of tumours*	
Low grade	27 (63%)
Intermediate grade	5 (11.5%)
High grade	11 (25.5%)
	
*Stage*	
I	19 (44%)
II	6 (14%)
III	3 (7%)
IV	15 (35%)
	
*Primary treatment*	
Radical surgery	36 (83.7%)
Surgery and full-dose radiotherapy	7 (16.3%)
	
*Outcome*	
Alive and disease free	33 (76.8%)
Alive with disease	6 (13.9%)
Died of disease	4 (9.3%)

**Table 2:** Relationship between histological grade of tumours, EGFR gene copy number, EGFR expression and pERK1/2 expression with disease-free interval (DFI) and overall survival (OS)

**Variable**	**DFI**	**OS**
*Histological grade*		
3 *vs* 2+1	*P*=0.001	*P*=0.001
		
*EGFR gene copy number*		
Polysomy *vs* normal	*P*=0.003	*P*=0.019
		
*EGFR protein expression*		
Positive *vs* negative	*P*=0.286	*P*=0.307
		
*pERK1/2 expression*		
Positive *vs* negative	*P*=0.004	*P*=0.001

Abbreviations: EGFR=epidermal growth factor receptor  pERK1/2=activated extracellular signal-regulated kinases.
